# Ice Nucleation Activity and Aeolian Dispersal Success in Airborne and Aquatic Microalgae

**DOI:** 10.3389/fmicb.2018.02681

**Published:** 2018-11-12

**Authors:** Sylvie V. M. Tesson, Tina Šantl-Temkiv

**Affiliations:** ^1^Aquatic Ecology, Department of Biology, Faculty of Science, Lund University, Lund, Sweden; ^2^Section for Microbiology, Department of Bioscience, Aarhus University, Aarhus, Denmark; ^3^Department of Physics and Astronomy, Stellar Astrophysics Centre, Aarhus University, Aarhus, Denmark; ^4^Department of Bioscience, Arctic Research Centre, Aarhus University, Aarhus, Denmark

**Keywords:** airborne microalgae, aquatic microalgae, ice nucleation activity, heterogeneous ice nucleation, atmospheric deposition

## Abstract

Microalgae are common members of the atmospheric microbial assemblages. Diverse airborne microorganisms are known to produce ice nucleation active (INA) compounds, which catalyze cloud and rain formation, and thus alter cloud properties and their own deposition patterns. While the role of INA bacteria and fungi in atmospheric processes receives considerable attention, the numerical abundance and the capacity for ice nucleation in atmospheric microalgae are understudied. We isolated 81 strains of airborne microalgae from snow samples and determined their taxonomy by sequencing their ITS markers, 18S rRNA genes or 23S rRNA genes. We studied ice nucleation activity of airborne isolates, using droplet freezing assays, and their ability to withstand freezing. For comparison, we investigated 32 strains of microalgae from a culture collection, which were isolated from polar and temperate aqueous habitats. We show that ∼17% of airborne isolates, which belonged to taxa Trebouxiphyceae, Chlorophyceae and Stramenopiles, were INA. A large fraction of INA strains (over 40%) had ice nucleation activity at temperatures ≥-6°C. We found that 50% of aquatic microalgae were INA, but the majority were active at temperatures <-12°C. Most INA compounds produced by microalgae were proteinaceous and associated with the cells. While there were no deleterious effects of freezing on the viability of airborne microalgae, some of the aquatic strains were killed by freezing. In addition, the effect of desiccation was investigated for the aquatic strains and was found to constitute a limiting factor for their atmospheric dispersal. In conclusion, airborne microalgae possess adaptations to atmospheric dispersal, in contrast to microalgae isolated from aquatic habitats. We found that widespread taxa of both airborne and aquatic microalgae were INA at warm, sub-zero temperatures (>-15°C) and may thus participate in cloud and precipitation formation.

## Introduction

Airborne microalgae dispersal may have critical consequences for weather and climate (Knopf et al., 2010; Alpert et al., 2011), human and animal health (Genitsaris et al., 2011a) as well as urban and natural environment (Sahu and Tangutur, 2014). Terrestrial and marine microalgae are regularly aerosolized due to mechanical disturbances and get transported to new environments by atmospheric currents (Tesson et al., 2016). More than 353 morphological taxa of eukaryotic microalgae, including Chromista and Viridiplantae (Tesson et al., 2016), and a few genera of Cyanobacteria have been identified in the atmosphere (Sharma et al., 2007). Airborne microalgae can successfully colonize new terrestrial habitats, such as building surfaces and open water containers (Barberousse et al., 2006; Genitsaris et al., 2011b). Thus, atmospheric dispersal of microalgae impacts their biogeographic distribution, both on local and global scales, and may, in combination with the shifts in environmental conditions, promote the expansion of certain microalgal species. For instance, a freshwater raphidophyte *Gonyostomum semen* has progressively expanded through northern Europe during the past decades, which may partially be driven by aeolian dispersal (Lebret et al., 2015). Our limited knowledge of controls on microalgae dispersal through the atmosphere hampers our ability to predict changes in their biogeographical patterns.

An important factor for successful aeolian dispersal is the cell’s capacity to survive and reduce exposure to atmospheric conditions. Certain terrestrial and marine microalgae have properties that promote survival and activity in dessicated, frozen and hyperosmotic conditions, e.g., incorporated in sea-ice (e.g., Ewert and Deming, 2013) or snow (e.g., Mosser et al., 1977). As major atmospheric stressors are drying-rewetting and freeze-thawing cycles, adaptations to these stressors that were aquired by microalgae in terrestrial and marine habitats, may promote their successful aeolian dispersal. A long residence time in the atmosphere may additionally undermine dispersal success of airborne microalgae (e.g., Schlichting, 1964). The removal of airborne microalgae from the atmosphere is driven by dry and wet deposition. While dry deposition is strongly affected by microalgal cell size, wet deposition is stimulated by microorganisms, which possess cloud-condensation and ice nucleation properties that promote cell incorporation into rain droplets (Burrows et al., 2009; Amato et al., 2015). Thus, microorganisms that produce INA compounds affect their own deposition to new environments (reviewed in Tesson et al., 2016). It is not known whether airborne microalgal strains have an increased ability to resist atmospheric stress or whether they synthesize INA compounds that promote their dispersal.

It has been proposed that microalgae can influence atmospheric processes (Möhler and Hoose, 2011; Wilson et al., 2015). Heterogeneous ice nucleation in clouds is induced by specific atmospheric particles called ice nuclei and is a key process involved in cloud development and wet precipitation in temperate regions (Burrows et al., 2009; Hoose and Möhler, 2012). Variable primary biological atmospheric particles, such as bacteria, fungi and pollen, are strikingly efficient at promoting ice formation at high sub-zero temperatures up to -1°C (Hoose and Möhler, 2012). To the contrary, mineral dust and other inorganic particles mostly nucleate ice below -15°C (Kanji et al., 2017). Biogenic ice nucleation is catalyzed either by non-proteinaceous macromolecules produced by pollen (Pummer et al., 2015) or INA proteins synthesized by bacteria (Warren and Wolber, 1991; Šantl-Temkiv et al., 2015; Ling et al., 2018) and fungi (Lagzian et al., 2014). Several studies have confirmed that certain marine and sea-ice microalgae can nucleate ice at temperatures below -20°C (Sassen et al., 2003; Knopf et al., 2010; Alpert et al., 2011; Wilson et al., 2015). Diatoms could nucleate ice at temperatures <-23°C and their ice nucleation activity was associated with microalgal shells (Knopf et al., 2010; Alpert et al., 2011) or exudates (Wilson et al., 2015). Some microalgae could also nucleate ice at warmer subzero temperatures (>-7°C), and this activity was attributed to the synthesis of specialized INA proteins or the presence of epiphytic INA bacteria on microalgal cells (D’Souza et al., 2013; Kviìderovaì et al., 2013). Despite the evidence for ice nucleation by limited species of terrestrial and marine microalgae, the presence of ice nucleation activity in airborne microalgae remains equivocal.

To better understand the capacity of microalgae for aeolian dispersal, ice nucleation and deposition we (i) described the diversity of cultivable airborne microalgae associated with snow deposition; (ii) evaluated the capacity of airborne and aquatic microalgae for surviving freezing and desiccation (iii) determined the ice nucleation activity of cultivable airborne microalgae as compared to aquatic microalgae, (iv) identified the nature of molecules involved in their ice nucleation activity, and (v) investigated the capacity of airborne microalgae to proliferate in new environments after deposition.

## Materials and Methods

### Collection and Maintenance of Microalgal Strains

**FIGURE 1:**
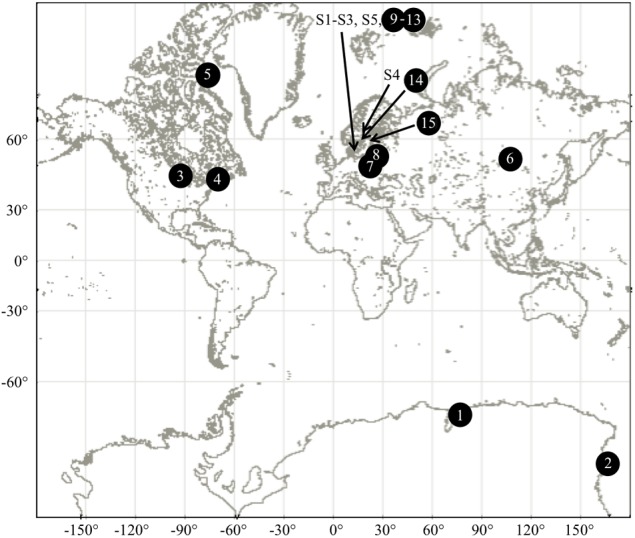
Sampling locations of aquatic and airborne strains of microalgae. See Supplementary Tables S1, S2 for further details.

We investigated 32 aquatic strains of microalgae from a culture collection available at Lund University, Department of Biology (Supplementary Table S1 and Figure 1) encompassing Cryptophytes, Raphidophytes, Dinoflagellates and Cyanobacteria. Strains were maintained in media mimicking their environment of origin, which was either freshwater (Modified Woods Hole Medium, MWC (Guillard and Lorenzen, 1972), brackish or marine water (f/2 25% and f/2 100% media, respectively (Guillard, 1975) using filtered 30 psu natural seawater). The light regime of 12 h light: 12 h dark, 50 μmol photon m^-2^ s^-1^ was used at either 4°C or 20°C (Supplementary Table S1).

Eighty-one airborne strains (Supplementary Table S2) were isolated from five atmospheric samples (Table 1). We collected samples from five snowfall events (Table 1) during winter 2016–2017 either directly into plastic containers thoroughly cleaned with ethanol 70% and rinsed with double distilled water or indirectly by sampling the upper 2 cm of freshly accumulated snow using sterile scrapers into a clean bucket. After sample collection, the snow was weighed and melted at 4°C in a controlled climate chamber. Melted snow was transferred to culture flasks (ca. 2–5 mL of sample per 40 mL final volume, 2–3 replicates per sample and medium) that mimicked four environmental conditions: artificial rain water (ARW) (Ekstrom et al., 2011), freshwater (MWC), brackish (f/2 25%), or saline (f/2 100%) environments. Enrichments were grown in a controlled culture chamber at 4°C under a 12 h:12 h light:dark regime, 50 μmol photon m^-2^ s^-1^. After approximately 2 months of growth, single cells were manually isolated using a micro-capillary pipet, under a CKX31SF Olympus microscope. Single cells were first washed in clean medium for three consecutive times and then transferred into 200 μL of the respective culture medium. The growth and purity of isolates was checked using a light microscope. Clean inoculum was then transferred into a 25 mL cell and tissue culture flask (VWR International, Lutterworth, United Kingdom) and maintained under the same culture conditions until further analyses.

**Table 1:** Information of the snow samples.

#	Date	Duration^AB^	Height^BC^	Depth^C^	Elevation^C^	Device^D^	Location	Weight^B^
S1	20160110	8 h	NA	NA	0.40 m	Bucket	Lund, Sweden	NA
S2	20160114	8 h	3.3 ± 0.25 cm	2 cm	12 m	Scraper	Lund, Sweden	0.6 Kg
S3	20160119	19 h	3.1 ± 0.23 cm	2 cm	12 m	Scraper	Lund, Sweden	2.8 Kg
S4	20160122	NA	21 ± 1 cm	3 cm	0 m	Scraper	Sundvall, Sweden	NA
S5	20160306	23 h	NA	NA	12.38 m	Bucket	Lund, Sweden	2.4 Kg


*^*A*^The duration of snowfall event prior to sample collection.*

*^*B*^NA stands for information not available.*

*^*C*^The deposited height and sampling depth of the snow cover was measured at each sampling location.*

*^*D*^Samples were collected either directly into the bucket or the upper 2 cm of snow were scraped off and stored.*

### Genetic Identification of Airborne Strains

Taxonomy of aquatic strains was previously reported (Logares et al., 2009; Rengefors et al., 2012; Pȩczuła et al., 2013; Lebret et al., 2015; Johansson et al., 2016; Karosienë et al., 2016) or obtained through personal communication. We identified the 81 isolated strains of airborne microalgae. For each strain, microalgal cells were collected using a two-step centrifugation: 25 mL culture was centrifuged at 3000 × *g* and 4°C for 15 min, the supernatant was removed and the pellet was suspended in 1.5 mL of medium, which was then centrifuged at 20,200 × g and 4°C for 10 min (Tesson et al., 2011). Genomic DNA (gDNA) was extracted using a CTAB protocol described in Panova et al. (2016). gDNA was quality controlled and quantified using three techniques: 1.5% agarose gel (TAE 1×) stained with GelRed^TM^ (10% final concentration, 41003, Biotium), a Nanodrop 2000 Spectrophotometer (Thermo Scientific) and a Qubit dsDNA Broad Range Kit (Q32853, dsDNA BR Assay Kit, Life Technologies Corp.).

The genetic markers used for inferring the taxonomy were internal transcribed spacer region (ITS) ITS1-5.8S-ITS2, a partial sequence of the gene encoding the small rRNA subunit (18S rRNA gene) or the gene encoding the 23S rRNA gene (23S rRNA gene). These markers were amplified using a polymerase chain reaction with 1.5 mM of MgCl_2_ (Applied Biosystems), 1× Buffer (10× PCR Buffer II, Applied Biosystems), 125 μM of each dNTP’s (Thermo Scientific), 0.4 μM of the forward and of the reverse primer, 0.5 U of AmpliTaq^®^ DNA-polymerase (Applied Biosystems) and 20–25 ng of genomic DNA in a final volume of 25 μL. The ITS region (ca. 700–800 bp) was amplified using the primer pair ITS-A (5′-GGG ATC CGT TTC CGT AGG TGA ACC TGC-3′) and ITS-B (5′-GGG ATC CAT ATG CTT AAG TTC AGC GGG T-3′) (Coleman et al., 1994) with an initial denaturation at 97°C for 5 min, followed by 30 cycles of 95°C for 1.25 min, 60°C for 2 min and 72°C for 4 min, and a final extension at 72°C for 7 min. The partial 18S rRNA gene (ca. 700–800 bp) was amplified using the primer pair EukA (5′-AAC CTG GTT GAT CCT GCC AGT-3′) (Medlin et al., 1988) and Euk1633rE (5′-GGG CGG TGT GTA CAA RGR G-3′) (Dawson and Pace, 2002) with an initial denaturation at 96°C for 2 min, followed by 26 cycles of 96°C for 1 min, 50°C for 1 min and 72°C for 2 min, and a final extension at 72°C for 7 min (modified from Genitsaris et al., 2009). The 23S rRNA gene (ca. 400-500 bp) was amplified using the primer pair p23SrV_f1 (5′-GGA CAG AAA GAC CCT ATG AA-3′) and p23SrV_r1 (5′-TCA GCC TGT TAT CCC TAG AG-3′) with an initial denaturation at 94°C for 2 min, followed by 35 cycles of 94°C for 20 s, 55°C for 30 s and 72°C for 30 s, and a final extension at 72°C for 10 min (Sherwood and Presting, 2007). PCR products were checked on a 1.5% agarose gel prior to purification. Ten microliter of amplicon was purified with 1 μL Exonuclease I (72073-Affymetrix Inc., Cleveland, OH, United States) and 2 μL FastAP Thermosensitive Alkaline Phosphatase (EF0651, Thermoscientific, Vilnius, Lithuania) and successively incubated at 37°C for 15 min and 85°C for 15 min. Purified PCR products were quantified using the Qubit method. Between one and three genetic markers were sequenced for each isolate (Supplementary Table S2). Samples were prepared for sequencing using BigDye Terminator v 3.1 Cycle Sequence Kit (Applied Biosystems, Warrington, United Kingdom) according to the manufacturer instructions and sequenced in house on a ABI3100xl Genetic analyzer (Applied Biosystems, Waltham, MA, United States).

Sequences were first quality controlled using ApE (A plastic Editor, version 2.0.47) and then cleaned, edited and merged using BioEdit (version 2.7.5., Hall, 1999). The alignments were performed to the nt/nr nucleotide database in GenBank to infer the taxonomy of each isolate. Taxonomy was provided for sequences that covered at least 89% of the query, with sufficiently low *e*-value (<e^-189^) and an identity >89%. Sequences were classified at the species level when the identity was at least 97% and at genus level for identity of at least 95% (as recommended by Ong et al., 2013). For strains with ambiguous taxonomy at the genus and species level, a higher taxonomic level is presented.

### Droplet Freezing Assays

Ice nucleation activity of microalgal strains and the freezing profiles of INA strains were determined using a droplet-freezing assay that was described by Ling et al. (2018). Sample preparation was performed on ice to avoid damaging the cold-adapted strains and to preserve their ice nucleation properties. To concentrate and wash the cells, 2–6 mL of initial culture was spun down at 3000–6000 rpm for 10 min at room temperature (Minispin Centrifuge, Eppendorf), the supernatant was removed and the pellet was suspended in 1 mL of 1× phosphate-buffered saline (PBS) (0.01 M Na_2_HPO_4_, 1.8 mM KH_2_PO_4_, 0.14 M NaCl and 2.7 mM KCl, pH 6.8). The wash step was repeated twice.

Screening for INA strains was performed using sixteen replicates of 20–30 μL droplets of PBS containing suspended microalgae in 384-well plates (VWR International, Lutterworth, United Kingdom). Each plate also contained 16 replicates of INA *Pseudomonas syringe* R10.79 cells (Šantl-Temkiv et al., 2015) suspended in PBS, which were used as a positive control, and 16 replicates of PBS, which served as a negative control. Plates were incubated for 30 min at each of the 1–2°C temperature intervals, through a temperature gradient from -2 to -28°C using an environmental climate chamber (Binder MK115, Tuttingen, Germany). The fraction of frozen wells was quantified visually at each temperature.

For each INA strain, its freezing profile was investigated using 10-fold dilution series of a cell suspension prepared in the same way as for screening assays. Thirty-two 20 μL replicates of each dilution were loaded into 384-well plates and the freezing assays were performed as with screening assays. The freezing profile was determined for three biological replicates, thus in total 96 replicates were analyzed for each strain. The frozen fraction of replicate wells at a given temperature and the number of ice nucleating particles (INP) were calculated following the equations from Vali (1971). The frozen fraction, which was determined as the number of frozen replicate wells out of the total number of wells investigated in a set of experiment (FF_sample_), was corrected by subtracting the background, i.e., the frozen fraction obtained for the negative control (FF_control_). The number of INP per droplet (N_n_) was calculated using a modified equation from Vali (1971) taking into account the initial concentration of microalgal cells (Equation 1). N_n_ stands for the concentration of INA compounds per microalgal cell; c stands for the concentration of microalgal cells [cells μL^-1^] in the droplet; and V for the droplet volume [μL].

(1) $$N_{n}=\frac{-\left(l\,n\left(1-\left[{FF}_{sample}-{FF}_{control}\right]\right)\right)}{cV}$$

The negative N_n_ values were removed from the dataset. We also only considered the strains for which all three measurements were above the detection limit. Analysis was performed with the R software version 3.3.2 (R Core Team, 2016) using the packages plyr (Wickham, 2011), ggplot2 (Wickham, 2009), and grid (Murrell, 2005).

We performed heat, filter, and antibiotic treatments to investigate the nature of the INA compounds produced by INA microalgae. The heating assays, which were used to distinguish proteinaceous and non-proteinaceous compounds (Christner et al., 2008), were carried out by incubating samples at 100°C for 10 min to denature proteins. We heat treated both the microalgal cells in culture medium and washed in PBS, as well as their exudates. The samples were filter treated using a polycarbonate membrane (0.22 μm pore size, Q-max syringe, Frisenette, Denmark) to remove the cells and cell fragments. The filtrate represents the soluble fraction released by cells into the medium and was investigated for soluble INA compounds. Finally, antibiotic treatments were applied to prepare axenic cultures of three aquatic strains (PGCCMP1383, PASP-03, and PASP-04), thus killing potential epiphytic INA bacteria and deciphering whether the ice nucleation activity was linked to microalgae or to the bacteria associated with the microalgae. Axenic cultures were prepared by adding 50 μL of a mixture of penicillin G, streptomycin sulfate and chloramphenicol (Popovsky and Pfiester, 1990) to 2.5 mL of cultures. After a 24 h incubation, a few cells were transferred to sterile medium. The absence of bacteria was confirmed on axenic cultures fixed in 2% formaldehyde, stained with DAPI (final concentration 10%, D9542-1MG Sigma-Aldrich, Steinheim, Germany), observed with a Labophot-2 fluorescence microscope (Nikon) and an Infinity 1 camera with Infinity Analyze Software (version 6.5.4., Lumenera Corporation).

For quantifying cell concentrations, the samples were fixed with 1% Lugol’s iodine and stored at 4°C in dark. FlowCAM (Fluid Imaging Technology Inc., United States) was used with the following settings (diameter: 2 to 15–1000 μm depending on the strain investigated, distance to nearest neighbor of 0, a sample volume of 0.5 mL, a flow rate of 0.15 mL min^-1^, and an auto-image rate of 17 frames per second) and the VisualSpreadSheet software (v.3.7.5).

### Effect of Freezing on Microalgal Survival

The capacity of microalgae to survive exposure to freezing was investigated for all 32 aquatic strains and selected 38 airborne strains (˜50% of all airborne strains) after performing the droplet freezing assays. Microtiter plates were sealed with an adhesive film (MicroAmp^®^Clear, Applied Biosystems, Warrington, United Kingdom) to avoid cross contamination between wells and placed in a culture chamber at 4°C. Each replicate sample was enriched with the respective culture medium (Supplementary Tables S1, S2) and incubated under conditions favoring microalgal growth (light regime of 12 h light:12 h dark, 50 μmolphoton.m^-2^s^-1^, 4°C or 20°C, Supplementary Tables S1, S2). Each culture was regularly monitored for growth (asexual reproduction) and movement using a CKX31SF Olympus microscope over a period of 16 weeks for aquatic microalgae and 31 weeks for airborne microalgae.

### Effect of Desiccation on Aquatic Strains

Cell integrity after desiccation was investigated in 17 aquatic microalgal strains (Table 2) using a neutral red stain (72210 Sigma-Aldrich) that penetrates the cell wall, concentrates in the lysosome and stains viable cells red. Droplets of 10 μL of healthy cultures were placed, in triplicate, in an open petri dish, dried in a fume hood on ice for a period of 2 h, which allowed all droplets to completely dry out. Dry cells were suspended in 10–20 μL of 20% neutral red solution made from respective medium and incubated in dark for an hour. After incubation, controls and treated droplets were observed under the microscope. Eleven replicates of sterile medium, which was stained in the same way, were used as negative controls to rule out the effect of background noise. Effect of drying was further investigated in 2 strains of *G. semen* (GSNO-10, GSPA-08) for different desiccation periods (10, 30, 120 min). This experiment was performed for 5–10 replicates of single cells.

**Table 2:** Survival of selected aquatic microalgal strains exposed to desiccation.

Strain	Motility after drying^A^	Growth after drying^B^	Cell wall damaged [%]^C^	N^D^
*Cryptomonas*	None	None	100	3 replicates
GS1103-12	/	/	92	36 single cells
GSDA-04	/	/	100	36 single cells
GSJE-09	/	/	97	36 single cells
GSNO-10	/	/	97	36 single cells
GSPA-08	/	/	100	36 single cells
GSPA-23	/	/	100	36 single cells
PABR-04	None	None	/	/
PACO-11	None	None	/	/
PAER-01	None	None	/	/
PASP-01	None	None	/	/
PASP-03	None	None	/	/
PASP-04	None	None	/	/
PGCCMP-1383	None	None	/	/
PGCCMP-2088	None	None	/	/
SHAB-T35	None	None	/	/
SHVE-74	None	None	/	/


*^*A*^The mobility of the organisms after a desiccation event was assessed for each strain.*

*^*B*^The backlash mark stands for not available data.*

*^*C*^The percentage of organism that presented cell wall damaged after the desiccation event was estimated for each strain.*

*^*D*^N stands for the number of replicates utilized per strain.*

The neutral red solution did not stain *P. aciculiferum* due to the intense brown pigmentation of the organism. Instead, we chose to perform revival tests on 11 strains of *P. aciculiferum* (Table 2). Cultures were placed into 24-well plates (Nunc^TM^ multidish, VWR International, Lutterworth, United Kingdom) and dried on ice in a fume hood for 2 h, which allowed all samples to completely dry. The dried cells were then suspended in the respective growth medium and incubated for 16 weeks. Each dried culture was monitored, and compared against a non-dried culture, for cell growth (asexual reproduction) and movement (swimming behavior) using a microscope (Table 2).

### Simulated Habitat Preferences in Airborne Microalgae for Dispersal

Aeolian dispersal occurs only if the airborne microalgal propagules can reproduce after their deposition (Ronce, 2007). A comparison of the microalgal diversity retrieved in enrichments was performed to infer habitat preference of identified taxa. The taxa showing growth in two or more media were classified as generalists, whereas those taxa growing in one medium were classed as specialists.

### Statistical Analyses

Statistical analyses were performed and figures were prepared using the free software environment R (R Core Team, 2016). Test for equality of proportions with continuity correction was applied in order to compare result of desiccation and a Kruskal-Wallis rank sum test complemented by a pairwise comparison test using Dunn’s-test was applied to compare the number of INP produced per cell in airborne and aquatic strains subjected to different treatments. For statistical analyses in R, the CRAN packages lawstat and PMCMR (Pohlert, 2014) were used.

### Nucleotide Accession Numbers

Sequences of airborne strains (Supplementary Table S2) are available in GenBank (MK005063-MK005126).

## Results

### Airborne Microalgae Diversity

**FIGURE 2:**
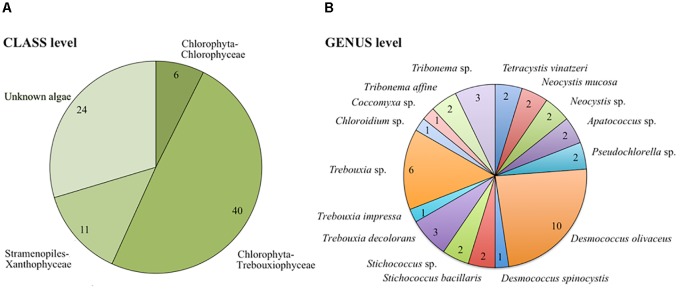
Taxonomic distribution of airborne communities at class **(A)** and genus/species **(B)** level. The number of strains is presented for each taxonomic category.

Out of 81 isolated airborne strains (Table 1), 56.8% were affiliated to chlorophyta, the majority of which were represented by the class Trebouxiophyceae (40 out of 46 strains) and the rest were affiliated to Chlorophyceae (6 out of 46 strains). Stramenopiles represented 13.6% of airborne strains and 29.6% belonged to unknown microalgae (Figure 2A and Supplementary Table S2). At the genus level, 42 strains were characterized (Figure 2B). Among Trebouxiophyceae, 35.5% of the strains belonged to *Desmococcus*, 32.3% to *Trebouxia*, 12.9% to *Stichococcus*, 6.5% to *Apatococccus*, 6.5% to *Pseudochlorella*, 3.2% to *Chloroidium*, and 3.2% to *Coccomyxa*. Among Chlorophyceae, we identified *Neocystis* (66.7%) and *Tetracystis* (33.3%). All the Xanthophytes (Stramenopiles) were represented by the genus *Tribonema*.

### The Effect of Subzero Temperatures and Freezing on Microalgae

About a third of the aquatic microalgae were able to survive exposure to -28 °C and freezing (10 out of 32), including five strains of *P. aciculiferum* (PABR-04, PACO-11, PASP-02, PASP-03, PASP-04), two strains of *Polarella glacialis* (PGCCMP-1383, PGCCMP-2088) and the three tested strains of *Apocalathium malmogiense* (Supplementary Table S1). These strains were able to recover growth by asexual reproduction and motility within the 4 months observation period. Further analyses would be necessary to identify the mechanisms that promoted the survival of these strains after freezing, e.g., production of anti-freeze proteins or exocellular polysaccharides. Frost damage had a detrimental effect on the majority of aquatic microalgae. For example, freezing caused loss of cell integrity, pigment, and motility in cells of *G. semen* and *Cryptomonas* sp. and in addition, prevented hatching of *G. semen* cysts into vegetative cells within 4 months. Freezing also inhibited proliferation of *Microcystis* sp. or *Peridinium baicalense*.

All of the 38 investigated airborne microalgal strains (Supplementary Table S2) were able to withstand freezing and resume growth within a week (98% ± 6% of revived replicates per strain, 16–96 replicates used per strain). Three of these strains were affiliated to Xanthophyceae (S4MWC-45, S4MWC-47, S5MWC-52), two strains to Chlorophyceae (*Tetracystis vinatzeri*; S3MWC-21, S3MWC-29) and 24 strains to Trebouxiophyceae (Supplementary Table S2) including one *Apatococcus* sp. (S2MWC-02), six *D. olivaceus*, two *Stichococcus* sp., nine *Trebouxia* sp. (including *T. decolorans* and *T. impressa*), and one *Coccomyxa* sp. (S3R-01). The remaining nine strains that were tolerant to freezing were unclassified (S2F4-01, S2F4-12, S3F4-64, S3R-02, S5MWC-02, S5MWC-23, S5MWC-56, S5MWC-58, S5MWC-65).

### The Effect of Desiccation on Aquatic Microalgae

Desiccation affected the survival of aquatic microalgae significantly (2-sample test for equality of proportions with continuity correction, *p* < 0.001, X-squared = 18.182, *df* = 1). Severe cell wall damage due to desiccation was observed in all Cryptomonas cells and in 92–100% of *G. semen* cells (Table 2). The effect of desiccation was equally detrimental for different exposure times in *G. semen* (GSNO-10 and GSPA-08), as all cells were damaged after 10 min of exposure. In some cases, a few cells maintained their cell integrity, but the population was not able to maintain motility or resume growth (Table 2). None of the 11 microalgal strains exposed to desiccation was able to resume growth by asexual reproduction or motility (Table 2) or form protective stages. Desiccation obstructed cyst hatching in cultures where cysts were present prior to the experiment. In addition to other damages, Cryptomonas cells exhibited loss of pigments and motility.

### Ice Nucleation Activity in Microalgae

**FIGURE 3:**
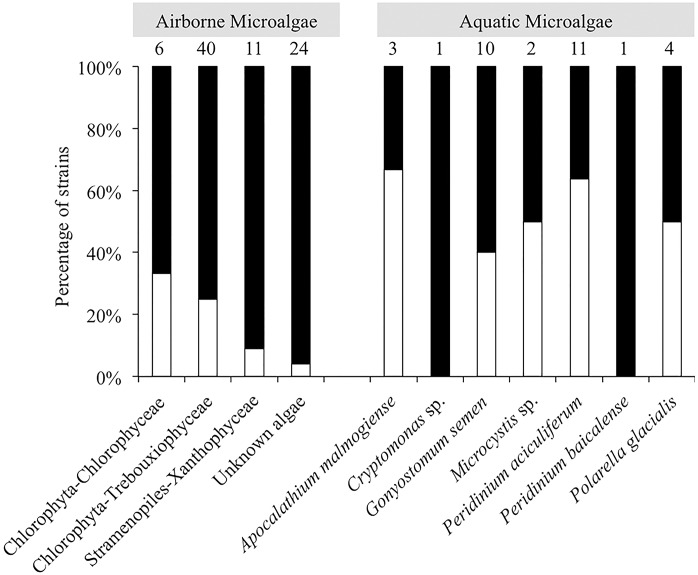
Percentage of airborne strains (class level) and aquatic strains (genus level) of microalgae that were INA (white bars) or were inactive (black bars).

In total, 66 strains of airborne microalgae out of 81 initially showed ice nucleation activity, which was defined as at least 50% wells frozen at -24°C. Repeated tests confirmed ice nucleation activity in 14 strains (17.3%, Figure 3). The six most active strains, which were INA ≥-6°C, were all affiliated to chlorophyta Trebouxiophyceae. Among the most active chlorophyta strains were *Trebouxia decolorans* S2RM-16 and S2RM-18, which initiated ice nucleation at -5°C with the production of 0.4–0.6 × 10^-2^ INP cell^-1^. Two INA chlorophyta strains affiliated to *Desmococcus olivaceus* (S2RM-21, S2RM-23) were capable of ice nucleation at -4°C with 0.2 × 10^-2^ - 0.2 × 10^-3^ INP cell^-1^. Similarly, two unknown strains of Trebouxiophyceae (S2RM-20 and S2RM-26) were INA at -5°C with the production of 0.5–0.1 × 10^-3^ INP cell^-1^. These microalgal strains produced few INP per cell that were active ≤-6°C, but could produce up to 0.3 INP cell^-1^ that were active at ≤-12°C.

Another five strains produced INP that were active between -6°C and -12°C, including *Trebouxia decolorans* S2RM-15 with 0.2 × 10^-2^ INP cell^-1^ at ≤-10°C, S5MWC-23 with 0.2 × 10^-2^ INP cell^-1^ at ≤-11°C, *D. olivaceus* S2RA-30 and S2RM-28 with 0.4–0.8 × 10^-3^ INP cell^-1^ at ≤-11°C, and *Stichococcus bacillaris* (S2RA-18) with 0.1 × 10^-3^ INP cell^-1^ at ≤-12°C. Two *Tetracystis vinatzeri* (S3MWC-21, S3MWC-29) were INA ≤ 12°C and produced 0.1–0.5 × 10^-2^ INP cell^-1^.

**FIGURE 4:**
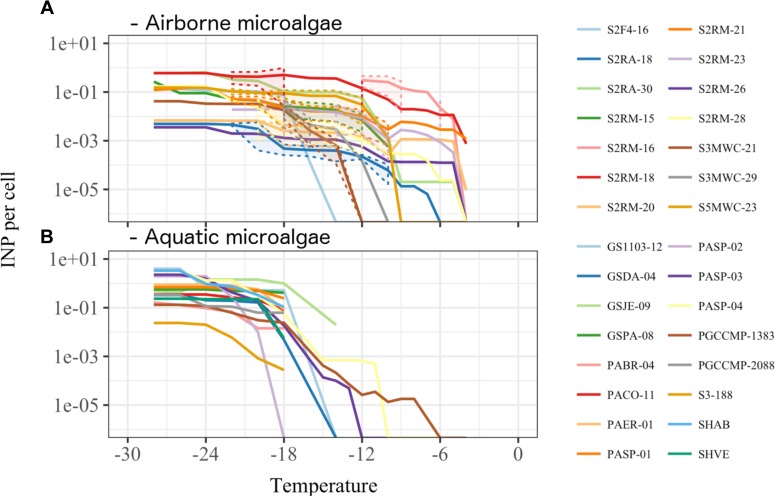
Ice nucleation activity estimated as number of ice nuclei per organism **(A)** for 14 airborne (Supplementary Table S2) and **(B)** for 16 aquatic microalgae (Supplementary Table S1). Dashed lines and shading in **(A)** indicate the standard deviation for the replicate measurements.

Among aquatic strains, 16 strains showed consistent ice nucleation activity. These included four strains of *G. semen*, seven strains of *Peridinium aciculiferum*, two strains of *Polarella glacialis*, one strain of *Microcystis* sp. and two strains of *A. malmogiense* (Figure 4 and Supplementary Table S3). The most active strains were *P. glacialis* PGCCMP-1383 and *P. aciculiferum* PASP-04 that produced INP active at ≤-7°C and ≤-11°C, producing 0.2 × 10^-4^ - 0.1 × 10^-3^ INP cell^-1^. Most aquatic strains were INA between -12°C and -18°C with 0.4 × 10^-2^ - 0.5 INP cell^-1^ (Figure 4 and Supplementary Table S3). *Microcystis* sp. S3-188 and *P. aciculiferum* PASP-02 started to nucleate ice in the temperature range of -18 to -24°C, with the production of 0.1 INP cell^-1^ in PASP-02 and 0.2 × 10^-2^ INP cell^-1^ for S3-188.

### Nature of the Component Responsible for Ice Nucleation Activity

**FIGURE 5:**
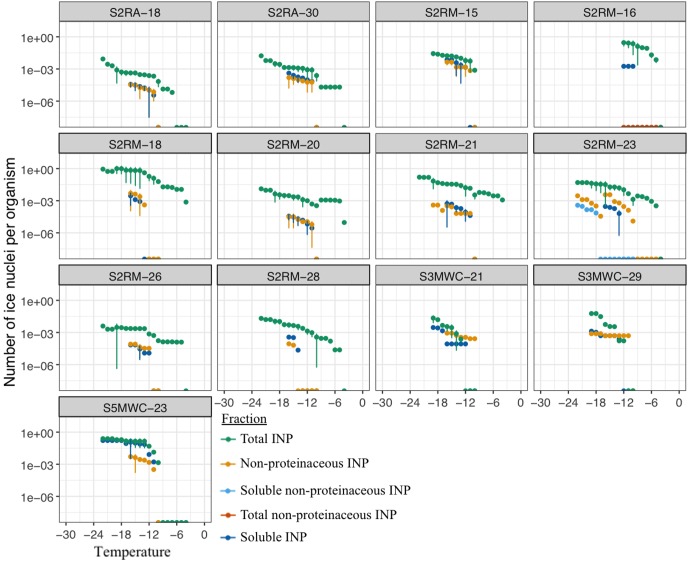
Freezing profiles for cells, heated cells and soluble compounds of 13 airborne microalgal strains (Supplementary Table S2).

Heat treatment was used to infer whether the INP produced by microalgae were proteinaceous. We found that all 13 airborne microalgal strains except for *Tetracystis vinatzeri* S3MWC-21 (Kruskal-Wallis chi-squared = 5.3, *df* = 2, *p* = 0.07; Dunn-test *p* = 0.35) produced INP that were likely of proteinaceous origin (Dunn-test *p* < 0.005) (Figure 5).

Filtration was used to investigate whether the particulate or the soluble fraction (<0.2 μm diameter) was responsible for the ice nucleation activity. The ice nucleation activity was significantly reduced in the soluble fraction in all airborne microalgal strains except in S3MWC-21 (Kruskal–Wallis chi-squared = 5.3, *df* = 2, *p* = 0.07; Dunn-test *p* = 0.08) and S5MWC-23 (Kruskal–Wallis chi-squared = 7.8, *df* = 2, *p* = 0.02; Dunn-test *p* = 0.77) (Figure 5). The freezing profiles of untreated and treated S3MWC-21 indicate that INA molecules were soluble, non-proteinaceous compounds released by the cells into their surroundings. In S5MWC-23, the INA molecules seem to be soluble and proteinaceous.

**FIGURE 6:**
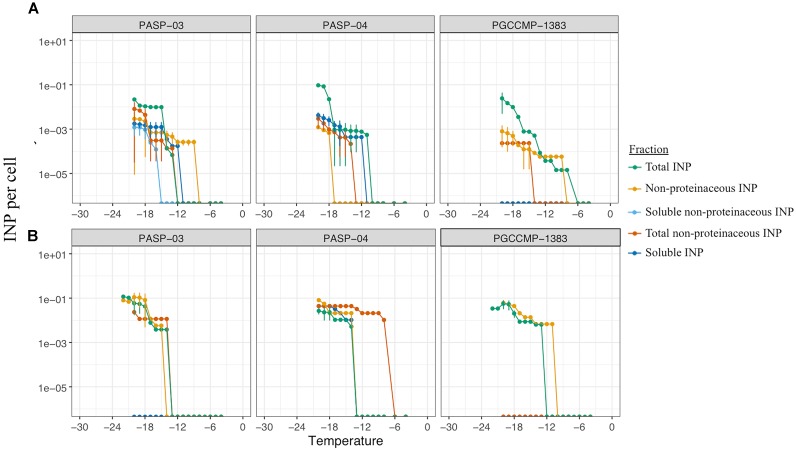
Freezing profiles to investigate the nature and fraction responsible of ice nucleation activity in three aquatic strains (Supplementary Table S1) under non-axenic **(A)** and axenic **(B)** conditions.

The characteristics of INA compounds was investigated in three aquatic microalgae. In PASP-03, the ice nucleation activity at temperatures <-16°C was reduced by filtration but not by heat treatment, indicating the INA molecules were non-proteinaceous compounds associated with the cells (Figure 6A). The ice nucleation compounds active ≥-18°C that were produced by PASP-04 were soluble and non-proteinaceous, whereas at temperatures ≤-18°C, the ice nucleation activity was due to INA compounds that were proteinaceous and associated with the cells (Figure 6A). The ice nucleation activity of PGCCMP-1383 was reduced by both the heat treatment and filtration treatment implying that the strain produced proteinaceous INA molecules that were associated with the cells (Figure 6A).

Antibiotic treatments produced axenic cultures of the three aquatic strains PASP-03, PASP-04, and PGCCMP-1383 and epibionts associated with microalgae were inactivated. All axenic cultures retained their ice nucleation activity, but their freezing profiles were altered (Figure 6B). While axenic cultures of PASP-03 and PGCCMP-1383 produced INP active at -14°C, axenic PASP-04 produced INP active at -8°C. In general, the concentration of soluble INP was reduced in axenic cultures. There was some variation in the nature of INP observed between axenic and non-axenic cultures, which implies that a combination of factors has an impact on the production and activity of INP.

We screened microalgal cells for the presence and survival of epibionts after antibiotic treatment and confirmed the absence of living epibionts associated with the microalgal cells (Supplementary Figure S1). In few cases, dead bacteria were visible (e.g., Supplementary Figures S1A,C). In PASP-04, extracellular lipopolysaccharides that previously formed the phycosphere remained associated with microalgae (Supplementary Figures S1C,D). Shells of dead microalgae were also visible as illustrated in Supplementary Figure S1F.

### Depositional Environment Preference in Airborne Microalgae

**FIGURE 7:**
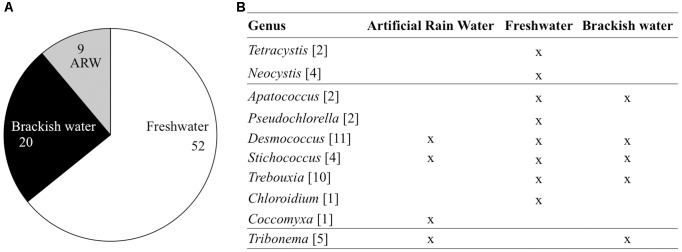
Depositional environment for the 81 airborne strains **(A)** and 42 strains identified down to genus level **(B)**. The number of representatives is indicated between brackets. ARW, Artificial Rain Water.

Three depositional environments were tested that simulated rain-forming puddles (artificial rain water), freshwater reservoirs (MWC) and brackish systems (f/2 25%). About 64% of the isolated airborne microalgae were isolated from media mimicking freshwater systems (Figure 7A), 25% of the strains from the brackish condition and 11% from the ARW. Half of the strains identified to genus level were able to grow in all three culture media. The other half of the genera were retrieved in only one medium type, e.g., *Pseudochlorella* sp., *Chloroidium* sp. and all chlorophyta grew only in freshwater medium and *Coccomyxa* sp. in rain forming puddles (Figure 7B). The more “generalist” strains, which exhibited the capacity to grow in different media after atmospheric dispersal, included *Desmococcus* sp. and *Stichococcus* sp. (Trebouxiophytes), *Trebouxia* sp. retrieved in fresh- and brackish waters, and *Apatococcus* sp. (Trebouxiophytes) and *Tribonema* sp. (Xanthophytes) retrieved in both rain water and brackish water systems (Figure 7B and Supplementary Table S2).

## Discussion

After emission of a microalgal cell into the atmosphere, its successful aeolian dispersal relies on its abilities to (1) maintain viability or form a persistent stage during its atmospheric residence time; (2) deposit to a new environment; and (3) reproduce in the new environment (Tesson et al., 2016). We demonstrate that numerous microalgal taxa are capable of dispersal through the atmosphere by studying the ability of selected aquatic and airborne microalgae to (i) tolerate desiccation, exposure to subzero temperature conditions and freezing; (iii) nucleate ice; and (iv) reproduce in diverse aquatic environments.

### The Diversity of Airborne Microalgae

We investigated the diversity of cultivable airborne microalgae that deposited with snow (Figure 2). The airborne microalgae were restricted to two phyla: the chlorophyta (Trebouxiphyceae and Chlorophyceae) and few Stramenopiles (Figure 2A and Supplementary Table S2). Many of the same airborne genera were detected in the atmosphere and on building surfaces by earlier studies (Tesson et al., 2016). However, atmospheric dispersal has previously not been shown for *Chloroidium* sp. *Neocystis* sp. and *Pseudochlorella* sp (Figure 2B and Supplementary Table S2). Genera identified from the snow samples contained strains originally described as terrestrial species (*Apatococcus, Chloroidium, Trebouxia, Tetracystis*, and *Stichococcus, Coccomyxa*), freshwater species (*Tribonema* and *Neocystis, Coccomyxa*) or associated with lichens (*Pseudochlorella*)^1^. Dispersal of Trebouxiphyceae, which represented ∼50% of all airborne strains may be promoted by its desiccation tolerance (Holzinger and Karsten, 2013). Furthermore, many other airborne strains belong to known terrestrial microalgae, which are frequently subjected to desiccation in terrestrial surface environments. Snow samples contained few aquatic microalgae, despite earlier reports of typically aquatic strains in the atmosphere. This discrepancy may be due to two reasons: first, we only studied the cultivable fraction of microalgal communities and second, we collected the precipitation samples during winter and outside of peak microalgae bloom periods. Overall, we found diverse airborne microalgae that are mostly associated with terrestrial environments in the winter precipitation samples.

### The Microalgal Capacity for Dispersal

We investigated the capacity of single-cell aquatic microalgae for atmospheric dispersal and found that desiccation was the major limiting factor for their survival, either causing severe cell wall damages or compromising their revival after deposition (Table 2). Even protective stages (e.g., pellicle cysts, resting stages) that normally promote microalgal survival under adverse conditions (Anderson and Wall, 1978) were negatively affected by desiccation (Table 2). This is in contrast with the observation of the wide geographical distribution of some of the investigated microalgal taxa, e.g., *G. semen* that recently expanded across lakes in Northern Europe with no water connectivity (Lebret et al., 2015; Sassenhagen et al., 2015). Variable modes of aerosolization and different drying rates may affect microbial cells differently, as desiccation of bacterial cells aerosolized through bubble bursting was found to promote the survival in comparison to surface drying (Alsved et al., in review). However, desiccation and low relative humidity have been previously shown to affect the metabolic activity and survival of microalgae within minutes to days (Ehresmann and Hatch, 1975; Davey, 2007; Wilson et al., 2015). Ehresmann and Hatch (1975) reported that direct exposure to air with relative humidity <91% was lethal for certain microalgae. We have not tested the airborne microalgae for desiccation tolerance, but we reason that their viability after airborne dispersal implies potential adaptation to atmospheric desiccation. In contrast, our study suggests that the feasibility of aquatic taxa for aeolian dispersal is reduced due to detrimental effects of desiccation.

The other major atmospheric stress that we investigated is the exposure to subzero temperatures and freezing. The consequences of such exposure are decreased rates of biochemical reactions, decreased membrane fluidity and cold denaturation of proteins (Fondi et al., 2016). In addition, formation of ice crystals can compromise cell wall integrity (Li et al., 1984; Price and Sowers, 2004; Pegg, 2010; Chen, 2015). While, all airborne microalgae could survive freezing and resume growth, only 31% of aquatic strains could withstand freezing even though many strains originate from polar regions (Supplementary Table S1). The freezing tests were performed using between five and several 100s of cells, which may imply that only a fraction of the population could recover after the exposure. Polar origin of aquatic strains did not always promote their freeze-tolerance. The two polar strains of *P. glacialis* isolated from marine environments could tolerate freezing, but *P. glacialis* strains from Antarctic lakes could not (Supplementary Table S1 and Figure 1). There are likely multiple mechanisms behind the observed tolerance of aquatic strains to subzero temperature and freezing. Protective mechanisms include production of anti-freeze proteins (Davies, 2014; Bar Dolev et al., 2016) and the ability to increase membrane fluidity and extracellular polysaccharides (Krembs et al., 2002; Wilson et al., 2015). Cold tolerance mechanisms were beyond the scope of this paper and not investigated. On the whole, our study showed that only few strains of aquatic microalgae can survive the conditions associated with atmospheric dispersal and may survive if aerosolized.

### Ice Nucleation Properties of Microalgae

We investigated the ability of microalgal strains to produce ice-nucleating compounds, which promote different forms of precipitation (Möhler et al., 2007). A large fraction of airborne and aquatic microalgae was found to nucleate ice between -5 and -24°C (Figures 3, 4 and Supplementary Table S3). While previous studies demonstrated ice nucleation activity in few microalgal strains (Worland and Lukešová, 2000; Knopf et al., 2010; Kviìderovaì et al., 2013; Wilson et al., 2015), we show for the first time that ice nucleation activity is common among variable taxonomic groups of microalgae. We also found that microalgae could nucleate ice at high subzero temperatures comparable to bacteria and fungi (Figure 4 and Supplementary Table S3). Ice nucleation activity was found to be strain specific. For example, while Kviìderovaì et al. (2013) reported ice nucleation activity in species of *Trebouxia* sp. (*T. asymmetrica, T. erici, and T. glomerata*) in the temperature range of -12 to -17°C, in our study *T. decolorans* (S2RM-15, S2RM-16, S2RM-18) was INA at temperatures ≤-5°C, and *T. impressa* (S4MWC-17) at temperatures ≤-24°C (Supplementary Table S3). In general, we showed that airborne microalgae were active at higher temperatures (>-12°C) than aquatic microalgae (<-12°C). A large proportion (17%) of viable microalgae that deposited through snow were INA (Figure 3). Due to their ice nucleation activity, these airborne microalgae likely have a higher probability of wet deposition, which reduces their atmospheric residence time and promotes their survival (Burrows et al., 2009; Amato et al., 2015). An even higher proportion of aquatic microalgae (∼50%) had the ability to nucleate ice (Figure 3). D’Souza et al. (2013) proposed that ice nucleation could be advantageous for aquatic diatoms. They found that an INA diatom species maintained its proximity to the photic zone by promoting frazil ice formation, thus increasing cell buoyancy (D’Souza et al., 2013). Overall, our study demonstrates that ice nucleation activity at temperatures higher than -15°C is common in both airborne and aquatic microalgae. The presence of INA microalgae in the atmosphere suggests that aside from bacteria and fungi, microalgae may also have an important role in cloud ice formation at high subzero temperatures.

The nature and size fraction of the INA molecules was studied in 14 airborne and 3 aquatic microalgae (Figures 4, 5). The majority of microalgae produced proteinaceous INA molecules that were not excreted into the surroundings. These INA molecules could either be produced by microalgae themselves, and possibly anchored into the microalgal shells, or by microbionts associated with the microalgal phycosphere such as INA bacteria or fungi (Lagzian et al., 2014; Ling et al., 2018). We found that a fraction of microalgae released non-proteinaceous or proteinaceous INA compounds into their surrounding (Figures 4, 5) which is in line with the findings of Wilson et al. (2015) and Ladino et al. (2016), who demonstrated INA exudates in marine diatoms. Fall and Schnell (1985) and D’Souza et al. (2013) isolated INA bacterial epibionts from the surface of microalgae and suggested that INA bacteria could be partially responsible for the ice nucleation activity in microalgae. To investigate the innate ability of microalgae to produce INP compounds, we produced three axenic aquatic microalgae cultures and confirmed that axenic cultures could produce INP comparable to non-axenic cultures (Figures 6A,B). Axenic cultures of PASP-03 and PGCCMP-1383 produce INP active at lower temperatures than the non-axenic cultures, which may imply either that epibionts were partially responsible for the ice nucleation activity or that the overall fitness of microalgal cells was reduced by the antibiotic treatment, which affected their ice nucleation activity (Supplementary Table S3). Microalgae, from which the phycosphere was removed, were earlier shown to keep INA (Worland and Lukešová, 2000; Kviìderovaì et al., 2013), however, the epibionts that are tightly associated with the algal shell were likely still present. Thus, this is to our knowledge the first study that shows ice nucleation activity in axenic strains of microalgae. In general, most airborne and aquatic INA microalgae produced proteinaceous INA compounds that were associated with the cells.

### The Ecological Specialization in Airborne Microalgae

Microalgae reproduction is required for successful dispersal and establishment in the depositional environment (Ronce, 2007). Deposition to an environment that supports colonization and reproduction is driven by chance. A generalist ecological strategy is advantageous for airborne microalgae dispersing through the atmosphere. We found that ∼50% of airborne microalgal strains were able to grow in media mimicking rain-forming puddles, freshwater reservoirs and brackish systems (Figure 7). Such generalists were affiliated to Trebouxiophyceae: *Desmococcus* sp. (e.g., *D. olivaceus)* and *Stichococcus* sp. (e.g., *S. bacillaris*). Most of the generalist genera were previously associated with dry surface environments (Barberousse et al., 2006; Hallmann et al., 2013; Holzinger and Karsten, 2013). *D. olivaceus* has been found extremely versatile and was recovered from the Antarctic (Broady and Ingerfeld, 1993) as well as from open sewage systems (Sivasubramanian, 2016). We found that some strains of *Trebouxia* sp., *Apatococcus* sp., and *Tribonema* sp. were able to grow in two of the environments (Figure 7). More restricted habitat requirements were observed in *Neocystis* sp., *Tetracystis* sp., *Pseudochlorella* sp., *Chloroidium* sp., and *Coccomyxa* sp., which showed a preference for just one of the conditions. Such narrow habitat preference may constitute a limitation and reduce the chance for successful dispersal. We have, however, tested only a limited selection of aqueous environments and may have underestimated the versatility of some strains. In addition, some genera, which we consider specialists, may only be present in one of the medium due to their lower frequency in the atmosphere. In conclusion, the majority of cultivable airborne microalgae were generalist, which is an ecological strategy that promotes their chances for successful establishment after deposition to new environments.

## Conclusion

Airborne strains of chlorophyta and a few strains of stramenopiles were able to survive exposure to subzero temperatures and freezing whereas such tolerance was restricted to few aquatic microalgal strains. Furthermore, a large number of microalgae promoted ice formation at temperatures >-15°C, through the production of proteinaceous components. A majority of airborne microalgae were generalists and could revive in environments mimicking rain-puddles, freshwater reservoirs and brackish environments. These microalgae, thus, show phenotypic traits that promote their survival during atmospheric dispersal, initiation of wet deposition, and colonization after deposition. These traits show that microalgae are capable of dispersal through the atmosphere and participating in cloud and precipitation formation.

## Author Contributions

ST conceived the idea, acquired and analyzed the data. ST and TŠ-T designed the experiments, interpreted the data and wrote the manuscript. The authors contributed critically to the drafts and gave final approval for publication. The authors agree to be accountable for the content of the work.

## Conflict of Interest Statement

The authors declare that the research was conducted in the absence of any commercial or financial relationships that could be construed as a potential conflict of interest.

## Footnotes

1 www.algaebase.org

## Supplementary Material

Figure S1 Three aquatic strains after antibiotic treatment and DAPI staining. PASP-03 **(A,B)**, PASP-04 **(C,D)** and PGCCMP-1383 **(E,F)**.

Table S1 Information on aquatic strains is presented: taxonomy at species level, sampling location (origin), the location on Figure 1 (#), culture medium (freshwater: MWC, brackish-water: f/2 25%, sea water: f/2 100%), growth temperature and the capacity of revival assessed over four months after freezing (Revival).

Table S2 Information on airborne strains. The taxonomy is presented at the Phylum-Class-Order-Species level when available. As genetic markers we used partial 23S rRNA gene, 18S rRNA gene and/or ITS and the amplicon length is indicated in brackets [bp]. The culture medium was either artificial rain water (ARW), freshwater (MWC) or brackish-water (f/2 25%). The capacity of revival was assessed over 31 weeks after freezing. Not amplified indicates that none of the genetic markers could be amplified despite an efficient DNA extraction.

Table S3 Mean ice nuclei produced per cell for each category of temperature tested in strains of airborne and aquatic microalgae. NA stands for data not available (all wells frozen).

## Abbreviations

ARW: Artificial rain water
INA: Ice nucleation active
MWC: Modified woods hole medium
PBS: Phosphate-buffered saline
