# Supplementary figures and images for: Ice Nucleation Activity and Aeolian Dispersal Success in Airborne and Aquatic Microalgae
Source: Front Microbiol. 2018 Nov 12;9:2681. doi: 10.3389/fmicb.2018.02681 (PMC6240693; doi:10.3389/fmicb.2018.02681)

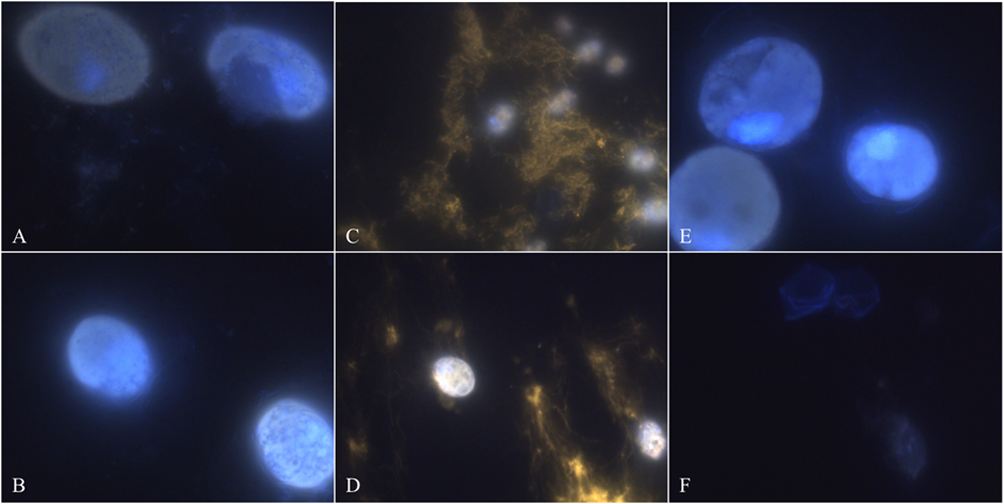

Supplement: Figure S1 — Three aquatic strains after antibiotic treatment and DAPI staining. PASP-03 (A,B), PASP-04 (C,D) and PGCCMP-1383 (E,F). [file Image_1.tif]
