# Supplementary material for: Ice Nucleation Activity and Aeolian Dispersal Success in Airborne and Aquatic Microalgae
Source: Front Microbiol. 2018 Nov 12;9:2681. doi: 10.3389/fmicb.2018.02681 (PMC6240693; doi:10.3389/fmicb.2018.02681)
Supplement: Table S1 — Information on aquatic strains is presented: taxonomy at species level, sampling location (origin), the location on Figure 1 (#), culture medium (freshwater: MWC, brackish-water: f/2 25%, sea water: f/2 100%), growth temperature and the capacity of revival assessed over four months after freezing (Revival). [file Table_1.pdf]

Table S1. Information on aquatic strains is presented: taxonomy at species level, sampling location (origin), the location on Figure 1 (#), culture medium (freshwater: MWC, brackish-water: f2 25%, sea-water: f2 100%), growth temperature (T) and the capacity of revival assessed over four months after freezing (Revival).

**Strain**	**Phylum**	**Species**	**Origin**	**#**	**Medium**	**T [°C]**	**Revival**
Cryptomonas	Cryptophytes	*Cryptomonas sp.*	Lake Highway, Antarctica [A]	1	F2 25%	4	no
GS1103-12	Raphidophytes	*Gonyostomum semen*	Minnesota, USA [B]	3	MWC	20	no
GSDA-04	Raphidophytes	*Gonyostomum semen*	Dagstorpssjön, Sweden [C]	13	MWC	20	no
GSJE-09	Raphidophytes	*Gonyostomum semen*	Jelino, Poland [D]	7	MWC	20	no
GSNO-10	Raphidophytes	*Gonyostomum semen*	Nobleboro, USA [E]	4	MWC	20	no
GSNO-12	Raphidophytes	*Gonyostomum semen*	Nobleboro, USA [E]	4	MWC	20	no
GSNO-18	Raphidophytes	*Gonyostomum semen*	Nobleboro, USA [E]	4	MWC	20	no
GSNO-28	Raphidophytes	*Gonyostomum semen*	Nobleboro, USA [E]	4	MWC	20	no
GSNO-35	Raphidophytes	*Gonyostomum semen*	Nobleboro, USA [E]	4	MWC	20	no
GSPA-08	Raphidophytes	*Gonyostomum semen*	Pabezdninkai, Lithuania [F]	8	MWC	20	no
GSPA-23	Raphidophytes	*Gonyostomum semen*	Pabezdninkai, Lithuania [F]	8	MWC	20	no
PABR-04	Dinoflagellates	*Peridinium aciculiferum*	Brodammen, Sweden [G]	10	MWC	4	yes
PABR-05	Dinoflagellates	*Peridinium aciculiferum*	Brodammen, Sweden [G]	10	MWC	4	no
PACO-11	Dinoflagellates	*Peridinium aciculiferum*	Copenhagen, Denmark [A]	9	MWC	4	yes
PAER-01	Dinoflagellates	*Peridinium aciculiferum*	Lake Erken, Sweden [G]	14	MWC	4	no
PAER-02	Dinoflagellates	*Peridinium aciculiferum*	Lake Erken, Sweden [G]	14	MWC	4	no
PAER-03	Dinoflagellates	*Peridinium aciculiferum*	Lake Erken, Sweden G	14	MWC	4	no

[A] Rengefors laboratory, [B] Lebret *et al.,* 2015, [C] Johansson *et al.,*2016, [D] Pęczuła, Poniewozik & Szczurowska 2013, [E] isolated by Rengefors K, [F] Karosienė *et al.,*2016, [G] Logares *et al.,*2009, [H] isolated by Logares R, [I] isolated by Björnerås C, [J] Rengefors, Logares & Laybourn-Parry 2012, [K]isolated by Kremp A. *Apocalathium malmogiense* previously referred as *Scrippsiella hangoei* (Craveiro et al.,2016).

PAER-08	Dinoflagellates	*Peridinium aciculiferum*	Lake Erken, Sweden [G]	14	MWC	4	no
PASP-01	Dinoflagellates	*Peridinium aciculiferum*	Stora Pildammen, Sweden [G]	11	MWC	4	no
PASP-02	Dinoflagellates	*Peridinium aciculiferum*	Stora Pildammen, Sweden [G]	11	MWC	4	yes
PASP-03	Dinoflagellates	*Peridinium aciculiferum*	Stora Pildammen, Sweden [G]	11	MWC	4	yes
PASP-04	Dinoflagellates	*Peridinium aciculiferum*	Stora Pildammen, Sweden [G]	11	MWC	4	yes
PB2-202a	Dinoflagellates	*Peridinium baicalense*	Lake Baikal, Russia [A]	6	MWC	4	no
PGAbrC	Dinoflagellates	*Polarella glacialis*	Lake Abraxas, Antarctica [G]	1	F2 100%	4	no
PGCCMP-1383	Dinoflagellates	*Polarella glacialis*	Ross Sea [G]	2	F2 100%	4	yes
PGCCMP-2088	Dinoflagellates	*Polarella glacialis*	Baffin Bay, Arctic [G]	5	F2 100%	4	yes
PGWI	Dinoflagellates	*Polarella glacialis*	Lake Williams, Antarctica [H]	1	F2 100%	4	no
S3-188	Cyanobacteria	*Microcystis sp.*	Lake Vombsjön, Sweden [I]	12	MWC	20	no
S4-16	Cyanobacteria	*Microcystis sp.*	Lake Vombsjön, Sweden [I]	12	MWC	20	no
SHAB-T35	Dinoflagellates	*Apocalathium malmogiense*	Lake Abraxas, Antarctica [J]	1	F2 25%	4	yes
SHLL-07	Dinoflagellates	*Apocalathium malmogiense*	Östersjön, Finland [K]	15	F2 25%	4	yes
SHVE-74	Dinoflagellates	*Apocalathium malmogiense*	Lake Vereteno, Antarctica [J]	1	F2 25%	4	yes

This is a provisional file, not the final typeset article

References

Craveiro, S.C., Daugbjerg, N., Moestrup, O. and Calado, A.J. (2016) Studies on *Peridinium aciculiferum* and *Peridinium malmogiense* (=Scrippsiella hangoei): comparison with *Chimonodinium lomnickii* and description of *Apocalathium gen. nov.* (Dinophyceae). Phycologia 56, 21-35. doi: 10.2216/16-20.1

Johansson, K.S., Luhrig, K., Klaminder, J. and Rengefors, K. (2016) Development of a quantitative PCR method to explore the historical occurrence of a nuisance microalga under expansion. Harmful Algae 56, 67-76. doi: 10.1016/j.hal.2016.04.012

Karosienė, J., Kasperovičienė, J., Koreivienė, J., Savadova, K. and Vitonytė, I. (2016) Factors promoting persistence of the bloom-forming Gonyostomum semen in temperate lakes. Limnologica 60, 51-58. doi: 10.1016/j.limno.2016.05.009

Lebret, K., Tesson, S.V.M., Kritzberg, E.S., Tomas, C. and Rengefors, K. (2015) Phylogeography of the freshwater raphidophyte *Gonyostomum semen* confirms a recent expansion in northern Europe by a single haplotype. J. Phycol. 51, 768-781. doi: 10.1111/jpy.12317

Logares, R., Boltovskoy, A., Bensch, S., Laybourn-Parry, J. and Rengefors, K. (2009) Genetic diversity patterns in five protist species occurring in lakes. Protist 160, 301-317. doi: 10.1016/j.protis.2008.10.004

Pęczuła, W., Poniewozik, M. and Szczurowska, A. (2013) Gonyostomum semen(Ehr.) Diesing bloom formation in nine lakes of Polesie region (Central–Eastern Poland). Ann. Limnol. - Int. J. Lim. 49, 301-308. doi: 10.1051/limn/2013059

Rengefors, K., Logares, R. and Laybourn-Parry, J. (2012) Polar lakes may act as ecological islands to aquatic protists. Mol. Ecol. 21, 3200-3209. doi: 10.1111/j.1365-294X.2012.05596.x
