# Supplementary material for: Ice Nucleation Activity and Aeolian Dispersal Success in Airborne and Aquatic Microalgae
Source: Front Microbiol. 2018 Nov 12;9:2681. doi: 10.3389/fmicb.2018.02681 (PMC6240693; doi:10.3389/fmicb.2018.02681)
Supplement: Table S2 — Information on airborne strains. The taxonomy is presented at the Phylum-Class-Order-Species level when available. As genetic markers we used partial 23S rRNA gene, 18S rRNA gene and/or ITS and the amplicon length is indicated in brackets [bp]. The culture medium was either artificial rain water (ARW), freshwater (MWC) or brackish-water (f/2 25%). The capacity of revival was assessed over 31 weeks after freezing. Not amplified indicates that none of the genetic markers could be amplified despite an efficient DNA extraction. [file Table_2.pdf]

Table S2. Information on airborne strains.

Strain	Phylum-Class[A]	Order-Species[A]	Amplicons[B]	Medium[C]	Revival[D]	Accession Number[E]
S1F4-04	Unknown algae	-	not amplified	F2 25%	NA	NA
S1F4-23	Unknown algae	-	not amplified	F2 25%	NA	NA
S1MWC-04	Chlorophyta-Trebouxiophyceae	*Prasiolales-Stichococcus bacillaris*	ITS(727)	MWC	NA	MK005063
S1MWC-08	Chlorophyta-Trebouxiophyceae	-	ITS(609),18S (620)	MWC	NA	MK005064, MK005120
S1MWC-09	Chlorophyta-Trebouxiophyceae	*Prasiolales*	ITS(734)	MWC	NA	MK005065
S1MWC-10	Chlorophyta-Trebouxiophyceae	*Trebouxiales-Trebouxia sp*	18S(346)	MWC	NA	MK005121
S2CENT-36	Chlorophyta-Trebouxiophyceae	*Trebouxiales-Trebouxia sp*	ITS(847)	MWC	yes	MK005066
S2CENT-37	Chlorophyta-Chlorophyceae	*Sphaeropleales-Neocystis mucosa*	ITS(207)	MWC	NA	MK005067
S2CENT-38	Chlorophyta-Chlorophyceae	*Sphaeropleales-Neocystis mucosa*	ITS(682)	MWC	NA	MK005068
S2CENT-44	Chlorophyta-Trebouxiophyceae	*Prasiolales-Desmococcus olivaceus*	ITS(735)	MWC	NA	MK005069
S2CENT-45	Chlorophyta-Trebouxiophyceae	*Trebouxiales-Trebouxia sp*	ITS(797)	MWC	yes	MK005070
S2CENT-56	Chlorophyta-Chlorophyceae	*Sphaeropleales-Neocystis sp*	ITS(660)	MWC	NA	MK005071
S2CENT-57	Unknown algae	-	not amplified	MWC	NA	NA
S2CENT-59	Chlorophyta-Chlorophyceae	*Sphaeropleales-Neocystis sp*	ITS(671)	MWC	NA	MK005072
S2CENT-61	Unknown algae	-	not amplified	MWC	NA	NA
S2CENT-62	Unknown algae	-	not amplified	MWC	NA	NA
S2F4-01	Unknown algae	-	not amplified	F2 25%	yes	NA

[A] The taxonomy is presented at the Phylum-Class-Order-Species level when available.
[B] As genetic markers we used partial 23S plastid gene, 18S and/or ITS rDNA genes and the amplicon length is indicated in brackets [bp]. Not amplified indicates that none of the genetic markers could be amplified despite an efficient DNA extraction.
[C] The culture medium was either artificial rain water (ARW), freshwater (MWC) or brackish-water (F2 25%).
[D] The capacity of revival was assessed over 31 weeks after freezing.
[E] NA stands for not available data.

S2F4-03	Chlorophyta-Trebouxiophyceae	*Prasiolales-Stichococcus sp*	ITS(627)	F2 25%	yes	MK005073
S2F4-12	Unknown algae	-	23S(332)	F2 25%	yes	MK005104
S2F4-15	Unknown algae	-	not amplified	F2 25%	NA	NA
S2F4-16	Stramenopiles-Xanthophyceae	*Tribonematales-Tribonema affine*	23S(409)	F2 25%	NA	MK005105
S2F4-19	Chlorophyta-Trebouxiophyceae	*Prasiolales-Desmococcus spinocystis*	ITS(570)	F2 25%	NA	MK005074
S2F4-21	Unknown algae	-	not amplified	F2 25%	NA	NA
S2F4-24	Stramenopiles-Xanthophyceae	*Tribonematales-Tribonema affine*	23S(402)	F2 25%	NA	MK005106
S2F4-25	Chlorophyta-Trebouxiophyceae	*Prasiolales-Desmococcus olivaceus*	ITS(760)	F2 25%	yes	MK005075
S2MWC-02	Chlorophyta-Trebouxiophyceae	*Chlorellales-Apatococcus sp*	ITS(752)	MWC	yes	MK005076
S2MWC-05	Chlorophyta-Trebouxiophyceae	*Chlorellales-Pseudochlorella sp*	ITS(634)	MWC	NA	MK005077
S2MWC-06	Chlorophyta-Trebouxiophyceae	*Chlorellales-Pseudochlorella sp*	ITS(633)	MWC	NA	MK005078
S2MWC-07	Unknown algae	-	not amplified	MWC	NA	NA
S2MWC-09	Chlorophyta-Trebouxiophyceae	*Prasiolales*	ITS(684)	MWC	NA	MK005079
S2MWC-11	Chlorophyta-Trebouxiophyceae	-	ITS(620),18S(603)	MWC	yes	MK005080, MK005122
S2MWC-12	Chlorophyta-Trebouxiophyceae	*Prasiolales*	ITS(562),18S(394)	MWC	NA	MK005081, MK005123
S2MWC-13	Chlorophyta-Trebouxiophyceae	*Prasiolales-Desmococcus olivaceus*	ITS(756)	MWC	NA	MK005082
S2MWC-15	Chlorophyta-Trebouxiophyceae	-	ITS(633),18S(618)	MWC	yes	MK005083, MK005124
S2MWC-16	Chlorophyta-Trebouxiophyceae	-	18S(421)	MWC	yes	MK005125
S2RA-18	Chlorophyta-Trebouxiophyceae	*Prasiolales-Stichococcus bacillaris*	ITS(422)	ARW	yes	MK005084
S2RA-21	Chlorophyta-Trebouxiophyceae	*Prasiolales-Stichococcus sp*	ITS(688)	ARW	NA	MK005085

S2RA-30	Chlorophyta-Trebouxiophyceae	*Prasiolales-Desmococcus olivaceus*	ITS(756)	ARW	yes	MK005086
S2RM-15	Chlorophyta-Trebouxiophyceae	*Trebouxiales-Trebouxia decolorans*	ITS(695)	MWC	yes	MK005087
S2RM-16	Chlorophyta-Trebouxiophyceae	*Trebouxiales-Trebouxia decolorans*	ITS(508),23S (386),18S(1541)	MWC	yes	MK005088, MK005107, MK005126
S2RM-18	Chlorophyta-Trebouxiophyceae	*Trebouxiales-Trebouxia decolorans*	23S(410)	MWC	yes	MK005108
S2RM-20	Chlorophyta-Trebouxiophyceae	-	23S(407)	MWC	yes	MK005109
S2RM-21	Chlorophyta-Trebouxiophyceae	*Prasiolales-Desmococcus olivaceus*	ITS(762)	MWC	yes	MK005089
S2RM-23	Chlorophyta-Trebouxiophyceae	*Prasiolales-Desmococcus olivaceus*	ITS(634)	MWC	yes	MK005090
S2RM-26	Chlorophyta-Trebouxiophyceae	*Prasiolales*	ITS(660)	MWC	yes	MK005091
S2RM-28	Chlorophyta-Trebouxiophyceae	*Prasiolales-Desmococcus olivaceus*	ITS(763)	MWC	yes	MK005092
S3F4-32	Chlorophyta-Trebouxiophyceae	*Prasiolales-Desmococcus olivaceus*	ITS(675)	F2 25%	NA	MK005093
S3F4-36	Unknown algae	-	not amplified	F2 25%	NA	NA
S3F4-37	Unknown algae	-	not amplified	F2 25%	NA	NA
S3F4-39	Unknown algae	-	not amplified	F2 25%	NA	NA
S3F4-46	Chlorophyta-Trebouxiophyceae	*Trebouxiales-Trebouxia sp*	ITS(723)	F2 25%	yes	MK005094
S3F4-50	Unknown algae	-	not amplified	F2 25%	NA	NA
S3F4-61	Chlorophyta-Trebouxiophyceae	*Chlorellales-Apatococcus sp*	23S(405)	F2 25%	NA	MK005110
S3F4-64	Unknown algae	-	not amplified	F2 25%	yes	NA
S3F4-66	Chlorophyta-Trebouxiophyceae	*Prasiolales-Desmococcus olivaceus*	ITS(762)	F2 25%	yes	MK005095
S3MWC-21	Chlorophyta-Chlorophyceae	*Chlamydomonales-Tetracystis vinatzeri*	ITS(608)	MWC	yes	MK005096
S3MWC-29	Chlorophyta-Chlorophyceae	*Chlamydomonales-*	ITS(609)	MWC	yes	MK005097

		Tetracystis vin atzeri				
S3R-01	Chlorophyta-Trebouxiophyceae	*Trebouxiophyceae ordo incertae sedis-Coccomyxa sp*	ITS(68)	ARW	yes	MK005098
S3R-02	Unknown algae	-	not amplified	ARW	yes	NA
S3R-11	Chlorophyta-Trebouxiophyceae	*Prasiolales-Desmococcus olivaceus*	ITS(496)	ARW	NA	MK005099
S4MWC-01	Unknown algae	-	not amplified	MWC	NA	
S4MWC-02	Stramenopiles-Xanthophyceae	-	23S(409)	MWC	NA	MK005111
S4MWC-07	Unknown algae	-	not amplified	MWC	NA	
S4MWC-10	Stramenopiles-Xanthophyceae	-	23S(409)	MWC	NA	MK005112
S4MWC-13	Chlorophyta-Trebouxiophyceae	*Trebouxiales-Trebouxia sp*	ITS(662)	MWC	yes	MK005100
S4MWC-17	Chlorophyta-Trebouxiophyceae	*Trebouxiales-Trebouxia impressa*	ITS(124)	MWC	yes	MK005101
S4MWC-18	Stramenopiles-Xanthophyceae	-	23S(409)	MWC	NA	MK005113
S4MWC-25	Chlorophyta-Trebouxiophyceae	*Trebouxiales-Trebouxia sp*	ITS(664)	MWC	yes	MK005102
S4MWC-45	Stramenopiles-Xanthophyceae	-	23S(322)	MWC	yes	MK005114
S4MWC-47	Stramenopiles-Xanthophyceae	-	23S(311)	MWC	yes	MK005115
S4MWC-51	Chlorophyta-Trebouxiophyceae	*Trebouxiophyceae ordo incertae sedis-Chloroidium sp*	ITS(578)	MWC	NA	MK005103
S5CARW-04	Stramenopiles-Xanthophyceae	*Tribonematales-Tribonema sp*	23S(409)	ARW	NA	MK005116
S5CARW-05	Stramenopiles-Xanthophyceae	*Tribonematales-Tribonema sp*	23S(409)	ARW	NA	MK005117
S5CARW-06	Stramenopiles-Xanthophyceae	*Tribonematales-Tribonema sp*	23S(409)	ARW	NA	MK005118
S5MWC-02	Unknown algae	-	not amplified	MWC	yes	NA
S5MWC	Unknown algae	-	not amplified	MWC	NA	NA

-22						
S5MWC -23	Unknown algae	-	not amplified	MWC	yes	NA
S5MWC -52	Stramenopiles-Xanthophyceae	-	23S(409)	MWC	yes	MK005119
S5MWC -56	Unknown algae	-	not amplified	MWC	yes	NA
S5MWC -58	Unknown algae	-	not amplified	MWC	yes	NA
S5MWC -65	Unknown algae	-	not amplified	MWC	yes	NA
