# Supplementary material for: Ice Nucleation Activity and Aeolian Dispersal Success in Airborne and Aquatic Microalgae
Source: Front Microbiol. 2018 Nov 12;9:2681. doi: 10.3389/fmicb.2018.02681 (PMC6240693; doi:10.3389/fmicb.2018.02681)
Supplement: Table S3 — Mean ice nuclei produced per cell for each category of temperature tested in strains of airborne and aquatic microalgae. NA stands for data not available (all wells frozen). [file Table_3.pdf]

Table S3. Mean ice nuclei produced per cell for each category of temperature tested in strains of airborne and aquatic microalgae. NA stands for not available data because all wells were already frozen.

Strain	[0°;-6°C[	[-6°;-12°C[	[-12°;-18°C[	[-18°;-24°C[	[-24°;-30°C[	Origin	Condition
S2F4-16	0.0000000	0.0000000	0.0000419	0.0971919	0.1282321	Airborne	non-axenic
S2RA-18	0.0000000	0.0000665	0.0003689	0.0019943	0.0049324	Airborne	non-axenic
S2RA-30	0.0000101	0.0008118	0.0857436	0.1745291	0.6185547	Airborne	non-axenic
S2RM-15	0.0000000	0.0019988	0.0181928	0.0361567	0.1494898	Airborne	non-axenic
S2RM-16	0.0038919	0.1537032	0.3077384	NA	NA	Airborne	non-axenic
S2RM-18	0.0061103	0.0380051	0.2451492	0.4094487	0.6011755	Airborne	non-axenic
S2RM-20	0.0004747	0.0008024	0.0023561	0.0050443	0.0068231	Airborne	non-axenic
S2RM-21	0.0020118	0.0054712	0.0147783	0.0406992	0.1439558	Airborne	non-axenic
S2RM-23	0.0001666	0.0024406	0.0122867	0.0196703	NA	Airborne	non-axenic
S2RM-26	0.0000627	0.0001834	0.0007331	0.0013159	0.0035653	Airborne	non-axenic
S2RM-28	0.0000119	0.0003828	0.0022807	0.0170734	0.1703241	Airborne	non-axenic
S3MWC-21	0.0000000	0.0000000	0.0013787	0.0254251	0.0391052	Airborne	non-axenic
S3MWC-29	0.0000000	0.0000000	0.0052935	0.0241139	NA	Airborne	non-axenic
S5MWC-23	0.0000000	0.0023123	0.0504848	0.0922745	0.1508952	Airborne	non-axenic
GS1103-12	0.0000000	0.0000000	0.4972322	0.6707031	3.5215115	Aquatic	non-axenic
GSDA-04	0.0000000	0.0000000	0.0080694	0.1221817	0.2851870	Aquatic	non-axenic
GSJE-09	0.0000000	0.0000000	0.0147091	1.2860438	0.9441319	Aquatic	non-axenic
GSPA-08	0.0000000	0.0000000	0.1307291	0.4614394	0.5644133	Aquatic	non-axenic
PABR-04	0.0000000	0.0000000	0.0179144	0.0322819	0.1339625	Aquatic	non-axenic
PACO-11	0.0000000	0.0000000	0.1189971	0.2565323	0.4742750	Aquatic	non-axenic
PAER-01	0.0000000	0.0000000	0.0841104	0.4900608	0.8823974	Aquatic	non-axenic
PASP-01	0.0000000	0.0000000	0.2429015	0.4581724	0.6956299	Aquatic	non-axenic
PASP-02	0.0000000	0.0000000	0.0000000	0.0966947	1.9669535	Aquatic	non-axenic
PASP-03	0.0000000	0.0000000	0.0075797	0.2141758	2.6787606	Aquatic	non-axenic
PASP-04	0.0000000	0.0001268	0.0175091	0.3521643	1.9737546	Aquatic	non-axenic
PGCCMP-1383	0.0000000	0.0000190	0.0036487	0.0345705	0.1404170	Aquatic	non-axenic
PGCCMP-2088	0.0000000	0.0000000	0.0387615	0.0930952	0.1934315	Aquatic	non-axenic
S3-188	0.0000000	0.0000000	0.0000000	0.0022292	0.0302583	Aquatic	non-axenic
SHAB	0.0000000	0.0000000	0.0802984	0.3963486	2.5440753	Aquatic	non-axenic
SHVE	0.0000000	0.0000000	0.0037146	0.2211920	0.3336112	Aquatic	non-axenic
PASP-03	0.0000000	0.0000000	0.0365990	0.0015928	NA	Aquatic	axenic
PASP-04	0.0000000	0.0000000	0.0068043	0.0031319	NA	Aquatic	axenic
PGCCMP-1383	0.0000000	0.0000000	0.0225206	0.0038930	NA	Aquatic	axenic
